# Development, *In-Vitro* Characterization and *In-Vivo* Osteoinductive Efficacy of a Novel Biomimetically-Precipitated Nanocrystalline Calcium Phosphate With Internally-Incorporated Bone Morphogenetic Protein-2

**DOI:** 10.3389/fbioe.2022.920696

**Published:** 2022-07-22

**Authors:** Gaoli Xu, Chenxi Shen, Haiyan Lin, Jian Zhou, Ting Wang, Ben Wan, Munerah Binshabaib, Tymour Forouzanfar, Guochao Xu, Nawal Alharbi, Gang Wu

**Affiliations:** ^1^ Department of Oral and Maxillofacial Surgery/Pathology, Amsterdam UMC and Academic Center for Dentistry Amsterdam (ACTA), Vrije Universiteit Amsterdam (VU), Amsterdam Movement Science (AMS), Amsterdam, Netherlands; ^2^ Department of Stomatology, Zhejiang Hospital, Hangzhou, China; ^3^ Hangzhou Huibo Science and Technology Co. Ltd., Xinjie Science Park, Hangzhou, China; ^4^ Department of Implantology, Hangzhou Stomatology Hospital, Hangzhou, China; ^5^ Savid School of Stomatology, Hangzhou Medical College, Hangzhou, China; ^6^ Department of Stomatology, Zhejiang Chinese Medical University, Hangzhou, China; ^7^ Department of Preventive Dental Sciences, College of Dentistry, Princess Nourah Bint Abdulrahman University, Riyadh, Saudi Arabia; ^8^ Department of Prosthetic Dental Sciences, King Saud University, Riyadh, Saudi Arabia; ^9^ Department of Oral Cell Biology, Academic Centre for Dentistry Amsterdam (ACTA), University of Amsterdam (UvA) and Vrije Universiteit Amsterdam (VU), Amsterdam, Netherlands

**Keywords:** nanocrystalline, hydroxyapatite, bone morphogenetic protein-2, biomimetic, bone regeneration

## Abstract

The repair of large-volume bone defects (LVBDs) remains a great challenge in the fields of orthopedics and maxillofacial surgery. Most clinically available bone-defect-filling materials lack proper degradability and efficient osteoinductivity. In this study, we synthesized a novel biomimetically-precipitated nanocrystalline calcium phosphate (BpNcCaP) with internally incorporated bone morphogenetic protein-2 (BpNcCaP + BMP-2) with an aim to develop properly degradable and highly osteoinductive granules to repair LVBDs. We first characterized the physicochemical properties of the granules with different incorporation amounts of BMP-2 using scanning electron microscopy, X-ray diffraction, Fourier transform infrared spectroscopy and X-ray photoelectron spectroscopy. We evaluated the cytotoxicity and cytocompatibility of BpNcCaP by assessing the viability and adhesion of MC3T3-E1 pre-osteoblasts using PrestoBlue assay, Rhodamine-Phalloidin and DAPI staining, respectively. We further assessed the *in-vivo* osteoinductive efficacy in a subcutaneous bone induction model in rats. *In-vitro* characterization data showed that the BpNcCaP + BMP-2 granules were comprised of hexagonal hydroxyapatite with an average crystallite size ranging from 19.7 to 25.1 nm and a grain size at 84.13 ± 28.46 nm. The vickers hardness of BpNcCaP was 32.50 ± 3.58 HV 0.025. BpNcCaP showed no obvious cytotoxicity and was favorable for the adhesion of pre-osteoblasts. BMP-2 incorporation rate could be as high as 65.04 ± 6.01%. *In-vivo* histomorphometric analysis showed that the volume of new bone induced by BpNcCaP exhibited a BMP-2 amount-dependent increasing manner. The BpNcCaP+50 μg BMP-2 exhibited significantly more degradation and fewer foreign body giant cells in comparison with BpNcCaP. These data suggested a promising application potential of BpNcCaP + BMP-2 in repairing LVBDs.

## 1 Introduction

Large-volume bone defects (LVBDs) can be resulted from congenital bone malformations, tumors, inflammation and trauma, severely compromising the aesthetics and functions of patients (Mansour et al., 2020; Migliorini et al., 2021). LVBDs override the self-healing capacity of bone tissue and thus do not heal spontaneously (Ma et al., 2021). Autologous bone grafts are still considered the gold standard to repair LVBDs in clinical practice since they contain osteoconductive scaffolds, osteoinductive growth factors and osteogenic cells (Kowalczewski and Saul, 2018). However, their application is associated with a series of drawbacks, such as limited availability, donor-site pain and morbidity as well as uncontrollable resorption rate (Baldwin et al., 2019). As alternatives, allogeneic and xenogeneic bone grafts are widely adopted as they bear bone-like structures and compositions (de Melo Pereira and Habibovic, 2018). Whereas, these materials are also associated with a series of concerns, such as disease transmission and severe immune reaction (Sohn and Oh, 2019). In this situation, synthetic calcium phosphate (CaP)-based bone grafts, such as tri-calcium phosphate (TCP) and hydroxyapatite (HA) show a promising application potential as they are available in unlimited quantities, free of pyrogens or antigens and also bear bone-like compositions (Piccinini et al., 2013). However, most of these materials do not bear intrinsic osteoinductivity so that they must be mixed with autologous bone grafts to heal LVBDs, in which the drawbacks of autologous bone grafts pursue (Vidal et al., 2020).

One viable method to confer osteoinductivity is to adopt an osteoinductive agent - bone morphogenetic protein-2 (BMP-2), a growth factor from the superfamily of transforming growth factor-β (TGF-β) (Wang et al., 2014). BMP-2 can induce *de novo* bone formation in ectopic sites, which proves its potent osteoinductivity. BMP-2 combined with collagen membrane has been approved for clinical applications to perform spine fusion, open fracture, anterior interbody fusion, and posterolateral lumbar fusion (Lo et al., 2012). Furthermore, BMP-2 may also be applied clinically to functionalize CaP-based bone grafts to promote bone regeneration. However, in order to achieve certain forms and mechanical stiffness, the production of most synthetic CaP bone grafts needs to be sintered at unphysiologically high temperatures (900–1,300°C) (de Groot, 1984; Layrolle and Daculsi, 2009; Diez-Escudero et al., 2020). Therefore, BMP-2 can only be absorbed superficially onto these bone grafts, for which BMP-2 will be burst released right after *in-vivo* implantation (Liu et al., 2018). The thereby-generated high concentration of BMP-2 may cause a series of side effects, such as osteolysis due to over-stimulated osteoclastic activity and bone formation in unintended sites (James et al., 2016).

One promising method to establish a CaP-based slow delivery system is biomimetic coating procedure, which is prepared in a physiological-like condition, such as 37°C and simulated body fluid (SBF) (Liu and Hunziker, 2009). BMP-2 may be co-precipitated into a thin layer of latticework of crystalline CaP on various substrates (Liu et al., 2010). In our previous study, we have shown that BMP-2-incorporated biomimetic CaP coating bore significantly higher osteoinductivity than the superficially adsorbed BMP-2 on various biomedical materials (Liu et al., 2006; Liu et al., 2007; Liu and Hunziker, 2009), such as titanium (Liu et al., 2007), polymers (Wu et al., 2010b) and deproteinized bovine bone (DBB) (Wu et al., 2011; Liu et al., 2013). Thereafter, we modified this protocol and realized a transition from a 2D coating to 3D granules. In our previous study, we have prepared a layer-by-layer (alternate amorphous CaP and crystalline CaP with about 3 cycles) assembled BioCaP-BMP-2 granules, which provides a slow-release carrier for BMP-2 (Zheng et al., 2014). In a recent study with a periodontitis model in beagle dogs, the co-administration of these granules with DBB was associated with significantly more connective tissue height, new cementum height, new bone height and area, as well as less down-growth of junctional epithelium than DBB alone (Wei et al., 2019). However, this type of granules bore insufficient mechanical strength and thus cannot be used alone as bone-defect-filling materials but only as an osteopromoter to enhance bone regeneration surrounding other bone-defect-filling materials, such as DBB.

To provide a viable option, in our previous studies, we have already presented a new type of CaP granules (Liu et al., 2017) that are fabricated using a modified protocol from our well-establish biomimetic coating technique (Liu et al., 2003; Liu et al., 2004; Wu et al., 2010a; Liu et al., 2018). CaP precipitates are achieved at 37°C in a Tris-HCL buffered five-fold supersaturated calcium phosphate solution and then subjected to overnight drying at room temperature. Such a fabrication in physiological temperature allows a co-precipitation of BMP-2 with CaP and a characteristic internal incorporation pattern of BMP-2 within the CaP granules, which enables a slow release of BMP-2 (Liu et al., 2005; Wu et al., 2010a; Liu et al., 2014; Liu et al., 2018). However, the thereby produced CaP granules are still suboptimal due to the simultaneous incorporation of chemicals, such as Tris base and sodium chloride, which may compromise the biocompatibility of the granules and the osteoinductive efficacy of BMP-2.

In this study, we further developed the protocol and fabricated a novel type of biomimetically-precipitated nanocrystalline calcium phosphate (BpNcCaP) granules with a minimized content of the unintended chemicals. We first characterized the physicochemical properties using scanning electron microscopy (SEM), X-ray diffraction (XRD), Fourier transform infrared spectroscopy (FTIR), X-ray photoelectron spectroscopy (XPS), and Vickers hardness (HV). We evaluated the cytotoxicity and cytocompatibility of BpNcCaP by assessing the viability and adhesion of MC3T3-E1 pre-osteoblasts using PrestoBlue assay, Rhodamine-Phalloidin and DAPI staining, respectively. We further assessed the *in-vivo* osteoinductive efficacy of the granules with different incorporation amounts of BMP-2 using micro-computed tomography (Micro-CT) and histomorphometric analysis.

## 2 Materials and Methods

### 2.1 Preparation of Granules

**FIGURE 1:**
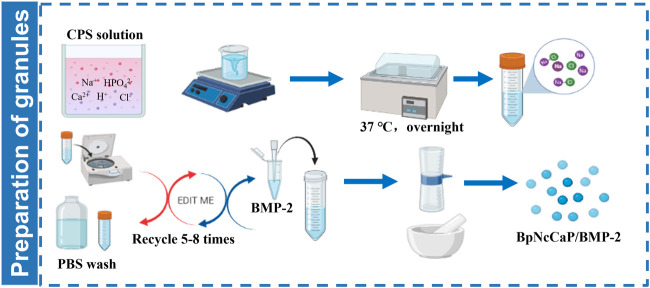
Schematic illustration of preparation of BpNcCaP granules with internally incorporated BMP-2.

Five-fold supersaturated CaP solution (200 mM HCl, 20 mM CaCl_2_·2H_2_O, 680 mM NaCl, 10 mM Na_2_HPO_4_, and 250 mM Tris [PH 7.4]) was incubated in a shaking water bath (50 agitations/min) at 37°C. After 24 h of incubation, the precipitation was retrieved by maximally removing the supernatant. Then the precipitates were subjected to centrifugation at a speed of 10,000 rpm/min for 10 min. The highly condensed precipitates were thoroughly washed by phosphate buffer saline (PBS) with an 8-fold volume of precipitates. Then the centrifugation/washing cycle was repeated so as to minimize the content of unintended chemicals. After the last centrifugation, BMP-2 solution (1.5 mg/ml, INFUSE^®^ Bone Graft, Medtronic, Minneapolis, MN, United States) at certain volumes was added to the precipitates to facilitate the incorporation of the target amount of BMP-2 as shown in Table 1. Then the BMP-2-containing precipitates were filtered using a top filter (0.22-μm pore, Corning, NY, United States) using a vacuum pump (−700 kPa) for 20 min to maximally remove the water content from the precipitates. Thereafter, the precipitates were dried overnight at room temperature in a safety cabinet until they were totally dry and hardened. Thereby achieved CaP blocks were carefully ground into small granules. Two sieves with pore diameters of 0.25 and 1 mm were used to retrieve the granules, whose sizes were ranging from 0.25 to 1 mm. All these fabrication procedures were performed in sterile conditions. The schematic illustration of preparation of BpNcCaP granules with internally incorporated BMP-2 was shown in Figure 1.

**TABLE 1:** The group set-up with the target dosage, actual dosage and corresponding loading efficiency of BMP-2 in the BpNcCaPs.

Groups (0.08 g/Sample)	Target Dosage of BMP-2/Sample (μg)	Actual Dosage of BMP-2/Sample (μg)	Loading Efficiency (%)
BpNcCaP	0	0	0
BpNcCaP+5 μg BMP-2	5	4.31 ± 0.17	43.08 ± 1.67
BpNcCaP+10 μg BMP-2	10	9.39 ± 0.14	46.97 ± 0.68
BpNcCaP+25 μg BMP-2	25	23.06 ± 2.19	57.65 ± 5.47
BpNcCaP+50 μg BMP-2	50	52.03 ± 4.81	65.04 ± 6.01

### 2.2 Determination of the Amount of the Incorporated BMP-2

The amount of incorporated BMP-2 was determined using an enzyme-linked immunosorbent assay (ELISA) kit (PeproTech, London, United Kingdom) as previously described (Zheng et al., 2014). 0.05 g of BpNcCaPs were dissolved in 1 ml 0.5 M Ethylenediaminetetraacetic acid (EDTA) (pH 8.0). The ELISA was performed according to the manufacturer’s instructions. Six samples were used for this purpose.

### 2.3 *In-vitro* Characterization

#### 2.3.1 Physical and Chemical Properties of BpNcCaP and BpNcCaP/BMP-2

The morphology of BpNcCaP without and with BMP-2 incorporations was examined by scanning electron microscopy (SEM) (Zeiss Sigma 300), the accelerating voltage was 3 kV, and the magnifications were ×5,000 and 50,000×. The grain size distributions of BpNcCaP and BpNcCaP+50 μg BMP-2 were analyzed using a software of Image-pro plus 6.0. The crystal structure of the synthesized BpNcCaPs was analyzed using X-ray diffraction (XRD) with a Siemens D5000 X-ray diffractometer (CuKα radiation, 40 kV and 30 mA). The XRD patterns were collected in the range of 2θ = 10–80° using 0.05° step and 2 s/step scan speed. The achieved experimental patterns were analyzed using Inorganic Crystal Structure Database (ICSD). Rietveld refinement was adopted to analyze the approximation of the XRD patterns of BpNcCaPs to the structural model of the Crystallographic Information File (CIF) available in crystallographic databases. VESTA (Visualization for Electronic and Structural Analysis) program was also used to simulate the unit cell structure of BpNcCaPs. The vibrational molecular spectra of BpNcCaPs were obtained from a Fourier transformed infrared (FTIR) spectrometer (Nicolet iS20, Thermo Scientific, United States) with a Diamond ATR accessory (Termo Scientific Instruments) in the spectral range from 4000 to 400 cm^−1^ wavenumbers. The elemental composition and chemical bonding of BpNcCaPs were obtained by X-ray photoelectron spectroscopy (XPS) using a spectrometer (K-alpha, Thermo Scientific, United States), a monochromatic Al Kα source was used as an X-ray source (hv = 1,486.6 eV), and high-resolution [O1s], [Ca2p] and [P2p] spectra were collected. The hardness was measured by a Vickers hardness tester (Falcon 507, Innovatest, Netherlands). During Vickers indentation, the load applied and holding time on the polished surface of BpNcCaP were 25 g and 10 s, respectively.

#### 2.3.2 Cytocompatibility of BpNcCaP

MC3T3-E1 pre-osteoblasts were obtained from the American Type Culture Collection (ATCC, Manassas, VA, United States) and cultured in alpha-modified Eagle’s medium (α-MEM; Welgene, Deagu, Korea) supplemented with 10% fetal bovine serum (FBS; Gibco, Carlsbad, United States), 100 U/mL penicillin, and 100 μg/ml streptomycin (GibcoTM, Thermo Fisher Scientific, United States), at 37°C in a 5% CO_2_ atmosphere. The medium was changed every 2 days.

Preparation of extracts of BpNcCaP and cell viability assay: Liquid extraction method has been recommended as a standard procedure to test cytotoxicity of biomedical materials in the ISO 10993. We mixed 250 mg of BpNcCaP with 5 ml of medium and incubated the suspensions at 37°C for 72 h. Control medium was prepared in the same manner without BpNcCaP. Suspensions were centrifuged at 1,500 rpm for 5 min and filtered through 0.22 µm membranes. Thereafter, the experimental concentrations (5, 10, 15, and 20 mg/ml) of BpNcCaP extracts were prepared with the medium using 50 mg/ml of the BpNcCaP extracts. MC3T3-E1 cells were seeded into 48-well plates at a density of 10^4^ cells/well in 5, 10, 15, and 20 mg/ml of BpNcCaP extracts and cultured for 0, 3, and 5 days 200 μl cell culture medium containing 10% PrestoBlue (Thermo Fisher Scientific, United States) solution was added to each well and the plates were incubated at 37°C for 1 h according to the manufacturer’s instruction. After incubation, 100 μl of PrestoBlue solution from each well of the assay plates (48-well plates) was transferred to a new well in 96-well plates and measured using a SpectraMax fluorescence multi-well plate reader with the excitation/emission wavelengths set at 560/590 nm.

Preparation of BpNcCaP discs and cell adhesion assay: We made the BpNcCaP round discs (diameter: 4 mm; height: 2 mm) using the same way as we made BpNcCaP granules in Section 2.1. BpNcCaP discs were put into 48-well plates (1 disc/well), then MC3T3-E1 cells were seeded on the discs at a density of 3 × 10^4^ cells/disc and cultured for 3 days. Cell/BpNcCaP samples were rinsed twice with PBS and fixed with PBS-buffered 4% paraformaldehyde for 30 min at room temperature. After washing three times with PBS, cell/BpNcCaP were incubated with 0.3% Triton-X 100 in PBS for 5 min. Then, actin filaments were stained by rhodamine-phalloidin (Actin-Tracker Red-555, Beyotime, China) and the cell nucleus was stained using DAPI (Beyotime, China). The cell/BpNcCaP samples were observed under a confocal laser scanning microscope (FV3000, Olympus, Japan).

### 2.4 *In vivo* Experiments

#### 2.4.1 Experimental Groups

In order to screen the optimal dosage of BMP-2, we set up BMP-2 at 5 μg, 10 μg, 25 μg and 50 μg per sample (0.08 g) of BpNcCaP granules as the target dosage. This dosage range of BMP-2 was set up according to our previous studies, in which tens micrograms of BMP-2 per sample is already sufficient to induce new bone formation (Hunziker et al., 2016; Wei et al., 2020). The experimental groups according to the target dosage of BMP-2 were defined as shown in Table 1. Five groups (*n* = 6 per group) were established: G1: BpNcCaP; G2: BpNcCaP+5 μg BMP-2; G3: BpNcCaP+10 μg BMP-2; G4: BpNcCaP+25 μg BMP-2; G5: BpNcCaP+50 μg BMP-2.

#### 2.4.2 Animal Surgery for Ectopic Subcutaneous Bone Formation

The animal experiment was approved by the Ethical Committee of Zhejiang Chinese Medical University (No. IACUC-20210104-02). Throughout the study, the SD rats were treated following the guidelines of animal care established by Zhejiang Chinese Medical University. Fifteen eight-week-old male SD rats were used as an animal model for ectopic subcutaneous bone formation (Wu et al., 2010b). For induction of a general anesthesia, 1% pentobarbital was intraperitoneally injected. The iliac crest was used as the landmark for determining the location of the skin incision, and a 25 mm posterior longitudinal incision was made bilaterally, 5–10 mm laterally from the midline. BpNcCaP groups were implanted into the subcutaneous space of the lumbar back according to a randomization protocol as used in our previous study (Wu et al., 2011). Right after implantation, the soft tissues were repositioned, and the wound was closed using standard non-resorbable suture materials. The wound was then disinfected with 10% povidone-iodine. Rats were kept at ambient temperature conditions until awakened.

### 2.5 Micro-Computed Tomography

All rats were sacrificed 5 weeks after surgery by intramuscular injection of excessive Sumianxin II. The implanted samples and surrounding tissues were harvested and immediately fixed in 10% neutral-buffered formalin for 2 days. The samples were then scanned using Micro-CT (µCT 100, Scanco Medical AG, Switzerland) at a resolution of 10 μm (80 kV, 100 µA) and then subjected to offline reconstruction. We evaluated the micro-architectures of bone using the following parameters: 1) Bone volume (BV, mm^3^); 2) Bone Mineral Density (BMD; mg HA/ccm); 3) Bone surface (BS, mm^2^) and 4) Structure model index (SMI).

### 2.6 Histology Procedures

After micro-CT analysis, the samples were rinsed in tap water, dehydrated in ethanol and embedded in methylmethacrylate as reported previously (Wu et al., 2010b; Wu et al., 2011). Samples were cut using a Leica diamond saw fitted in a Leica SP1600 slicing machine (Leica Microsystems, Wetzlar, Germany), the tissue blocks were cut into 5-7 slices, 300-µm-thick and 500 µm apart, according to a systematic random sampling protocol. All slices were glued to plastic specimen holders and ground down to a final thickness of 80–100 µm. They were surface-polished and surface-stained with McNeal’s Tetrachrome, basic Fuchsine and Toluidine blue as previously described (Wu et al., 2010b; Wu et al., 2011). The volume of the newly formed bone, blood vessels, undegraded particles and foreign body giant cells (FBGCs) were measured using the point-counting technique according to our previous publications (Liu et al., 2017; Teng et al., 2020).

### 2.7 Statistical Analysis

All data are presented as mean ± standard deviation (SD). Data were compared using one-way analysis of variance (ANOVA), and Bonferroni’s correction was used for post-hoc comparison. The significance level was set at *p* < 0.05.

## 3 Results

### 3.1 Characterizations of BpNcCaPs

#### 3.1.1 Surface Morphology of BpNcCaPs

**FIGURE 2:**
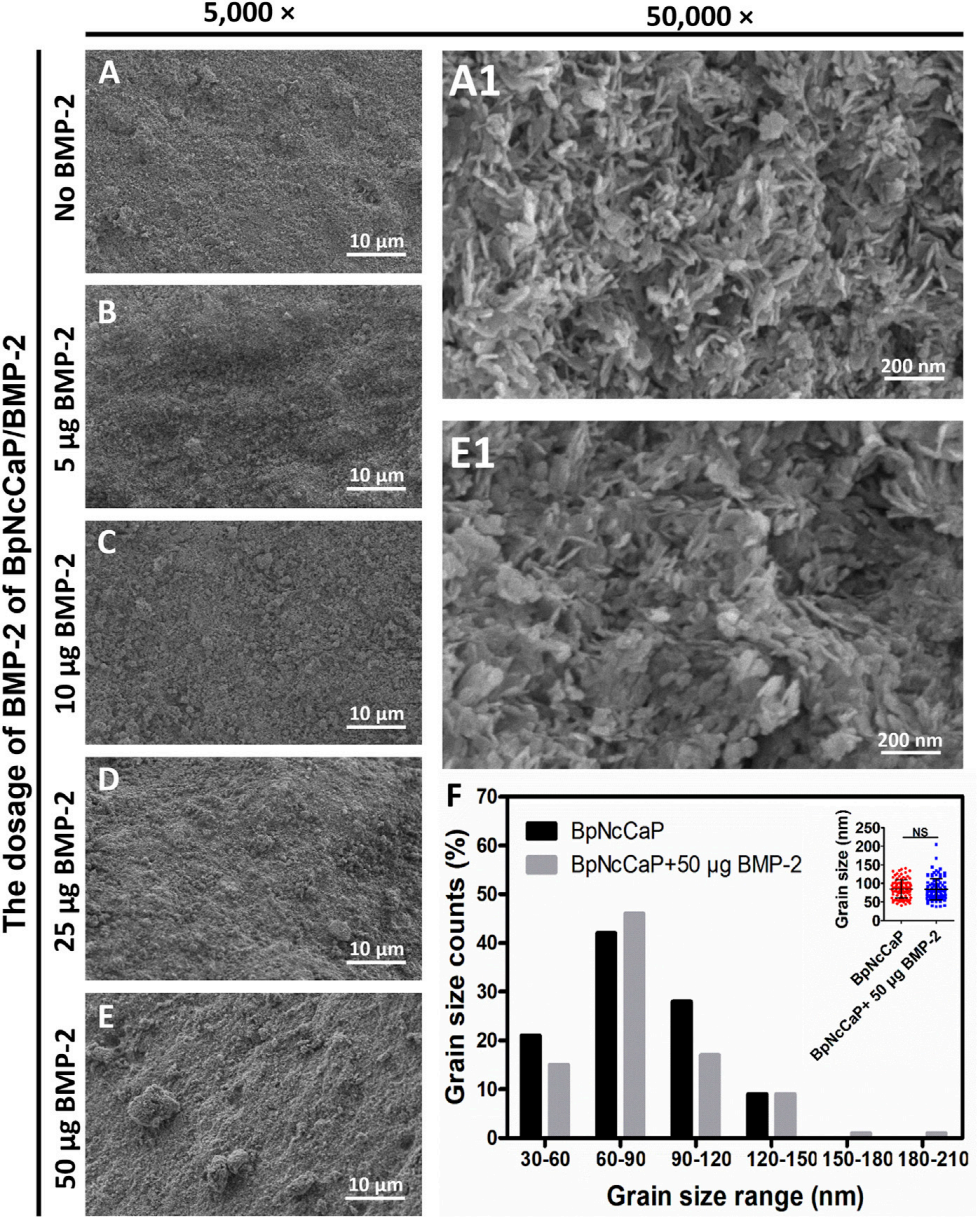
Scanning electron microscopy (SEM) observations of surface characteristic and morphology of BpNcCaP without or with different amounts (5/10/25/50 μg) of BMP-2. Representative SEM images of BpNcCaP without **(A)** or with different amounts (5/10/25/50 μg) of BMP-2 **(B–E)** in a lower magnification (×5,000). Bar = 10 μm. Higher-magnification (50,000×) SEM images of BpNcCaP **(A1)** and BpNcCaP+50 μg BMP-2 **(E1)**. Bar = 200 nm. **(F)** Graph depicting the grain size distribution of BpNcCaP and BpNcCaP+50 μg BMP-2.

Low-magnification SEM images showed that BpNcCaP bore a slightly rough and uneven morphology (Figure 2). This morphology was not changed by the presence of various amounts of BMP-2. High-magnification SEM images showed that BpNcCaP (Figure 2A1) and BpNcCaP+50 μg BMP-2 (Figure 2E1) were mainly comprised of needle-like crystals. The grain length distribution was shown in Figure 2F. The grain lengths of the crystals for BpNcCaP and BpNcCaP+50 μg BMP-2 were about 85.14 ± 24.48 nm and 84.13 ± 28.46 nm, respectively. The incorporation of 50 μg BMP-2 did not significantly change the morphology and the grain size.

#### 3.1.2 Crystallinity and Phase Composition of BpNcCaPs

**FIGURE 3:**
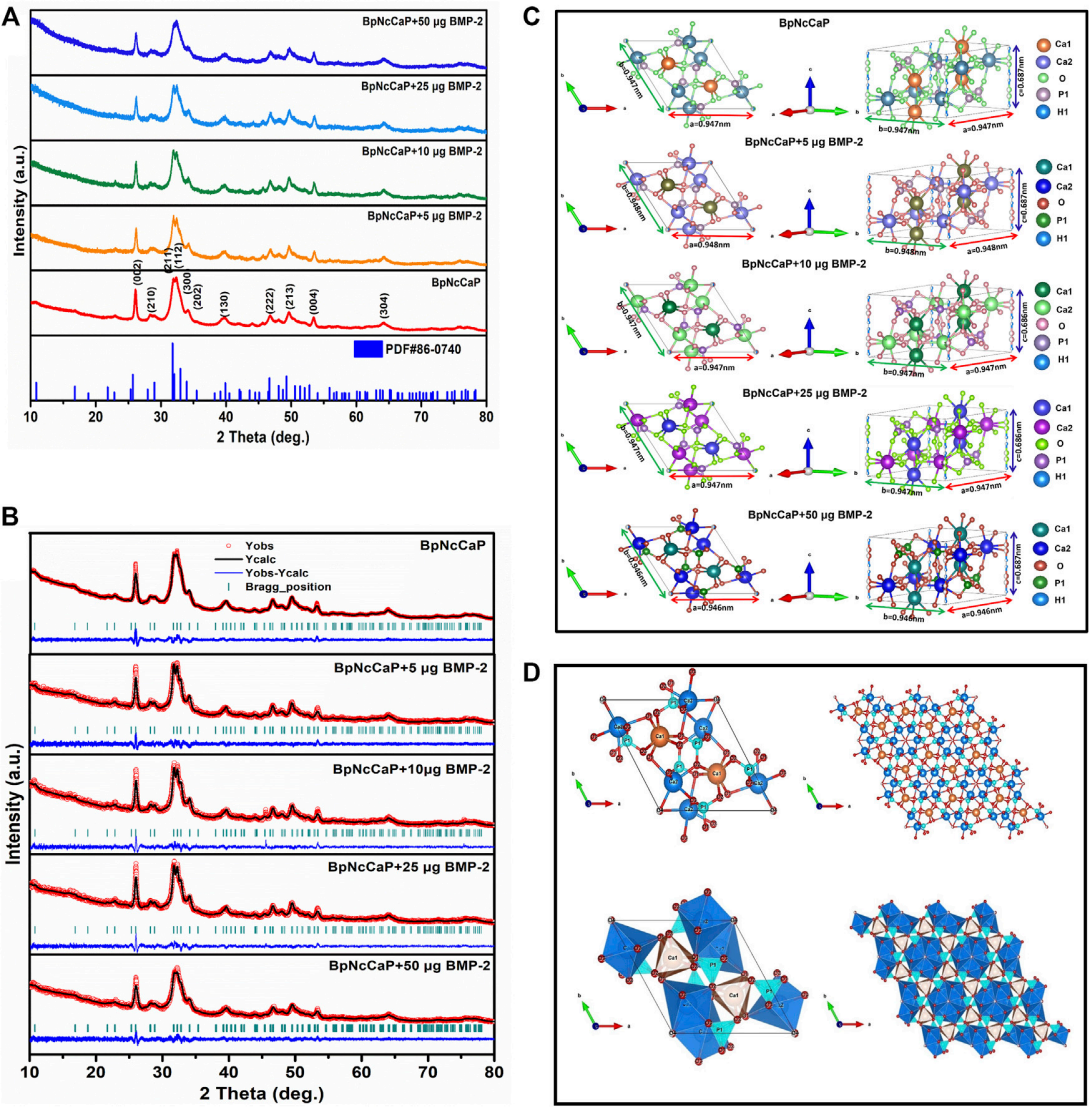
**(A)** XRD patterns of BpNcCaPs without or with 5/10/25/50 μg BMP-2 as well as the standard ICSD no. 86-0740. **(B)** Rietveld refinement of XRD patterns for BpNcCaP without or with different amounts (5/10/25/50 μg) of BMP-2. Black lines: XRD patterns; Red circles: Rietveld refined results; Blue curves: The differences between the XRD patterns and Rietveld refinement results; Green bars: The calculated Bragg peak positions. **(C)** Representation of the unit cells of BpNcCaPs without or with different amounts (5/10/25/50 μg) of BMP-2. **(D)** Visualization of structures of ball-and-stick and polyhedral models along with the c-axis for BpNcCaP.

We performed XRD analysis to identify the phases and crystallinities of the BpNcCaP without or with various amounts of incorporated BMP-2. The peaks were observed at around 2θ = 25.87°, 29.15°, 31.97°, 32.27°, 33.16°, 34.17°, 40.11°, 46.95°, 49.62° and 53.19°(Figure 3A), which, according to the ICSD-PDF card 86-0740, were the typical reflections of the respective crystalline planes (002), (210), (211), (112), (300), (202), (130), (222), 213) and (004) of hexagonal hydroxyapatite (HA). These characteristic peaks were not significantly influenced by the incorporation of BMP-2. The crystallite sizes were calculated as 24.6, 20.6, 25.1, 19.9, and 19.7 nm for BpNcCaP, BpNcCaP+5 μg BMP-2, BpNcCaP+10 μg BMP-2, BpNcCaP+25 μg BMP-2 and BpNcCaP+50 μg BMP-2, respectively (Table 2).

**TABLE 2:** Unit cell parameters, crystallite sizes and crystallinities of BpNcCaPs without or with different amounts (5/10/25/50 μg) of BMP-2.

Groups	Unit Cell Parameters				Crystallite Size	Crystallinity
	a(Å)	b(Å)	c(Å)	V(Å^3^)	Å	(%)
BpNcCaP	9.4643(4)	9.4643(4)	6.8666(3)	532.66(4)	246	92.27
BpNcCaP+5 μg BMP-2	9.4753(4)	9.4753(4)	6.8653(3)	533.79(4)	206	92.05
BpNcCaP+10 μg BMP-2	9.4683(5)	9.4683(5)	6.8609(4)	532.67(5)	251	94.07
BpNcCaP+25 μg BMP-2	9.4711(4)	9.4711(4)	6.8640(3)	533.22(4)	199	91.12
BpNcCaP+50 μg BMP-2	9.4590(6)	9.4590(6)	6.8655(4)	531.98(5)	197	92.12

Thereafter, we adopted the Rietveld refinement to analyze the approximation of the XDR patterns of BpNcCaPs to the structural model of the Crystallographic Information File (CIF) available in crystallographic databases for HA. Furthermore, we also used this analysis to investigate the crystalline phase fractions of BpNcCaP without or with BMP-2 incorporation. The calculated patterns (red line in Figure 3B) were adjusted to the observed experimental pattern (black line in Figure 3B). The observed and calculated XRD profiles of BpNcCaP without or with incorporated BMP-2 as well as the difference between both profiles (YObs-YCal) were shown in Figure 3B. All the diffraction peaks were perfectly indexed to the ICSD card 86-0740 (hexagonal lattice system and space group P63/m). The refined lattice parameters of BpNcCaP unit cell were a = b = 0.946 nm and c = 0.687 nm. Regardless of the incorporation amount, the presence of BMP-2 did not significantly change the lattice parameters (Table 2). With the network parameters (a, b, c, α, β, and γ) and atomic positions (x, y and z) obtained after Rietveld refinement, we further adopted the VESTA (Visualization for Electronic and Structural Analysis) program (Momma and Izumi, 2011) to simulate the unit cell structure of BpNcCaPs (Figures 3C,D). All these data indicated that BpNcCaP bore a hexagonal-type HA structure.

#### 3.1.3 Surface Spectra of BpNcCaPs

**FIGURE 4:**
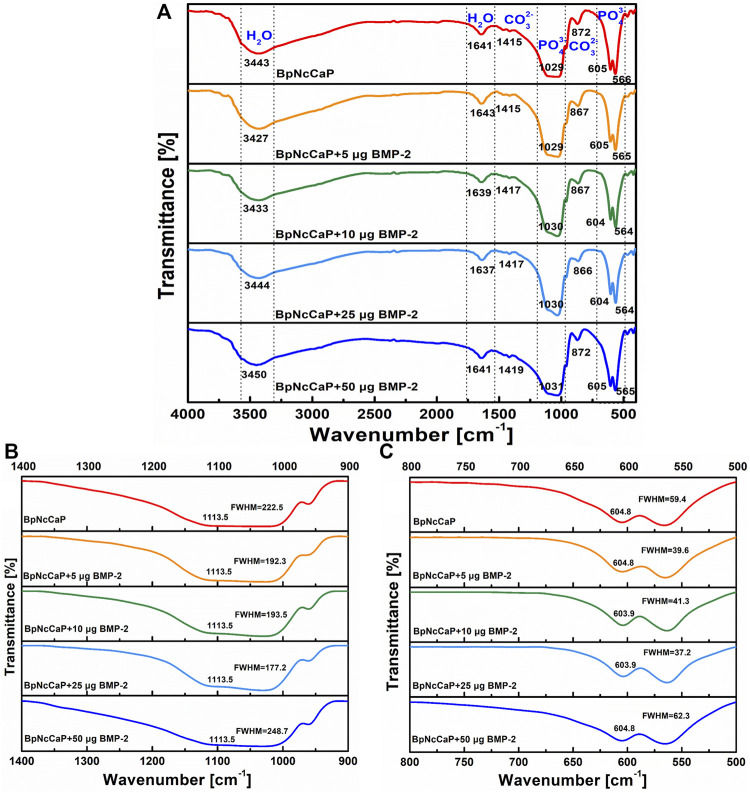
Total FTIR spectra **(A)**, FTIR spectra focusing on ν3 phosphate stretch in the 900–1,400 cm^−1^ wavenumber range **(B)**, and FTIR spectra focusing on ν4 bend in the 500–800 cm^−1^ wavenumber range **(C)** for BpNcCaPs without or with different amounts (5/10/25/50 μg) of BMP-2.

The FTIR spectra of BpNcCaP without or with the incorporation of BMP-2 were presented in Figure 4. The most prominent peak for BpNcCaP was the ν3 asymmetric stretching mode of the PO_4_^3−^ group with maxima at 1,029 cm^−1^. This peak shifted gradually to 1,031 cm^−1^ with the increase of BMP-2 amount. Another characteristic peak was the ν4 bending mode of PO_4_^3−^ with doublets at 564–566 cm^−1^ and 604–605 cm^−1^ for all BpNcCaP with different amounts of BMP-2. The full widths at half-maxima (FWHM) of the major ν3 band gradually decreased from 222.5 cm^−1^ in BpNcCaP to 192.3 cm^−1^ in BpNcCaP+5 μg BMP-2, 193.5 cm^−1^ in BpNcCaP+10 μg BMP-2, 177.2 cm^−1^ in BpNcCaP+25 μg BMP-2 (Figure 4B). In contrast, in BpNcCaP+50 μg BMP-2, the FWHM increased to 248.7 cm^−1^. A similar pattern was also found for the ν4 bending mode of PO_4_^3−^ (Figure 4C).

The weak bands of BpNcCaPs at 1,415–1,419 cm^−1^ and 866–872 cm^−1^ might be assigned to carbonate. Carbonates are constituents of bone structures, whose presence may improve the bioactivity of HA (Vallet-Regi and González-Calbet, 2004; Hoang et al., 2020). According to the literature (Fahami et al., 2012; Ignjatović et al., 2019; Edén, 2021), the functional groups generally observed in the FTIR spectra of CaP-based materials are PO_4_^3-^, OH^−^ and CO_3_^2-^ groups in the range 4000–300 cm^−1^. The phosphate group (PO_4_^3-^) appeared at around 1,100–1,019 cm^−1^ for the ν3 mode, 958 cm^−1^ for the ν1 mode, 605–530 cm^−1^ for the ν4 mode, and 500–400 cm^−1^ for the ν2 mode. Furthermore, three modes of OH^−^ ions, i.e., stretching, vibrational, and translational modes were at 3700-2500, 630, and 390 cm^−1^, respectively (Markovic et al., 2004; Alqap and Sopyan, 2009). The FTIR spectra of PO_4_^3-^, OH^−^ and CO_3_^2-^ groups of BpNcCaPs were identical to those of HA (Rodríguez-Lugo et al., 2018; Senthilkumar et al., 2021).

#### 3.1.4 Surface Composition of BpNcCaPs

**FIGURE 5:**
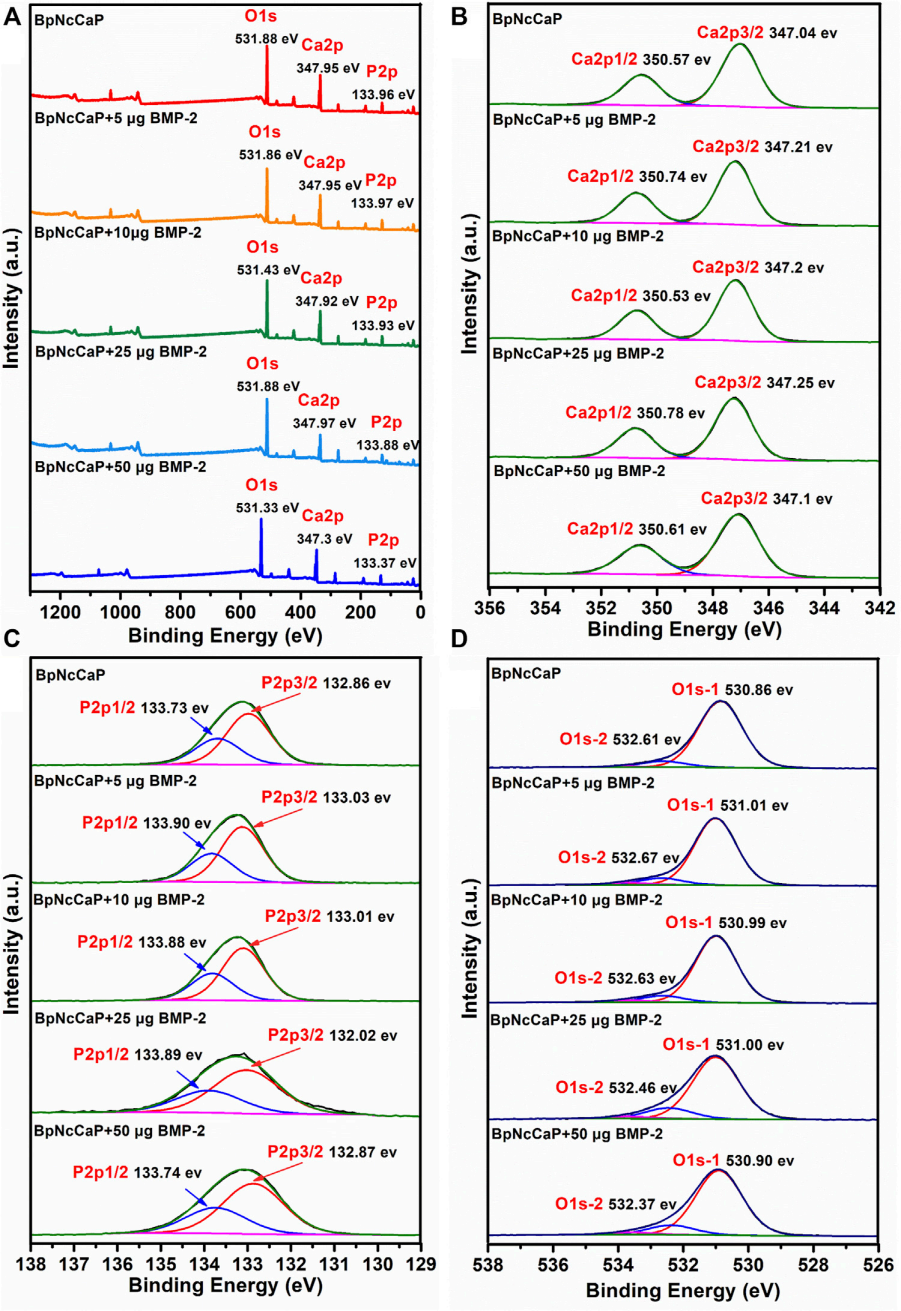
Total XPS survey spectra **(A)**, XPS spectra of Ca 2p **(B)**, XPS spectra of P 2p **(C)** and XPS spectra of O 1s **(D)** for BpNcCaPs without or with different amounts (5/10/25/50 μg) of BMP-2.

Figure 5A showed the XPS survey spectra of BpNcCaP without and with BMP-2 incorporation, consisting of the photoelectron peaks corresponding to oxygen (O), calcium (Ca) and phosphorus (P). These peaks corresponded to the HA (Rojas-Mayorga et al., 2016; Gomes et al., 2017). A detailed analysis of these XPS results was performed. The Ca 2p peak for BpNcCaP had been deconvoluted into two peaks: Ca 2p3/2 peak at 347.04 eV and Ca 2p1/2 peak at 350.57 eV (Figure 5B). These signals indicated the interaction Ca-POx. The P 2p spectrum had been deconvoluted into two peaks P 2p3/2 at 132.86 eV and P 2p1/2 at 133.73 eV (Figure 5C). These energies could be associated with P-O bonds from the phosphate group. The oxygen spectra (O 1 s) for BpNcCaP showed two peaks at 530.86 and 532.61 eV (Figure 5D). The O 1 s signal at 530.86 eV corresponded to O-C or P-O bonds, while the peak at 532.61 eV could be attributed to C-O groups. These findings were consistent with XPS results reported for Ca 2p, P 2p, and O 1 s in HA (Rojas-Mayorga et al., 2016; Gomes et al., 2017).

After BMP-2 incorporation, Ca 2p peaks at 347.30–347.97 eV, P 2p peaks at 133.37–133.97 eV, O 1s peaks at 531.33–531.88 eV for BpNcCaP + BMP-2 groups. The Ca 2p, P 2p, O 1s energy levels of BpNcCaP at 347.95, 133.96 and 531.88 eV shifted to lower energy values of 347.30, 133.37 and 531.33 eV of BpNcCaP+50 μg BMP-2, respectively (Figure 5A). And other binding energy changes in XPS spectra of BpNcCaPs were identified in the region of Ca 2p, P 2p, and O 1 s (Figures 5B–D), which suggested an interaction between BMP-2 with BpNcCaP. Regardless of the incorporation amount, the presence of BMP-2 did not significantly change the composition of BpNcCaP.

#### 3.1.5 Hardness of BpNcCaP

We adopted Vickers hardness test method to detect the hardness of BpNcCaP granules and added the data in Supplementary Figure S1. The vickers hardness of BpNcCaP was 32.50 ± 3.58 HV 0.025.

### 3.2 Cytotoxicity and Cytocompatibility of BpNcCaP

**FIGURE 6:**
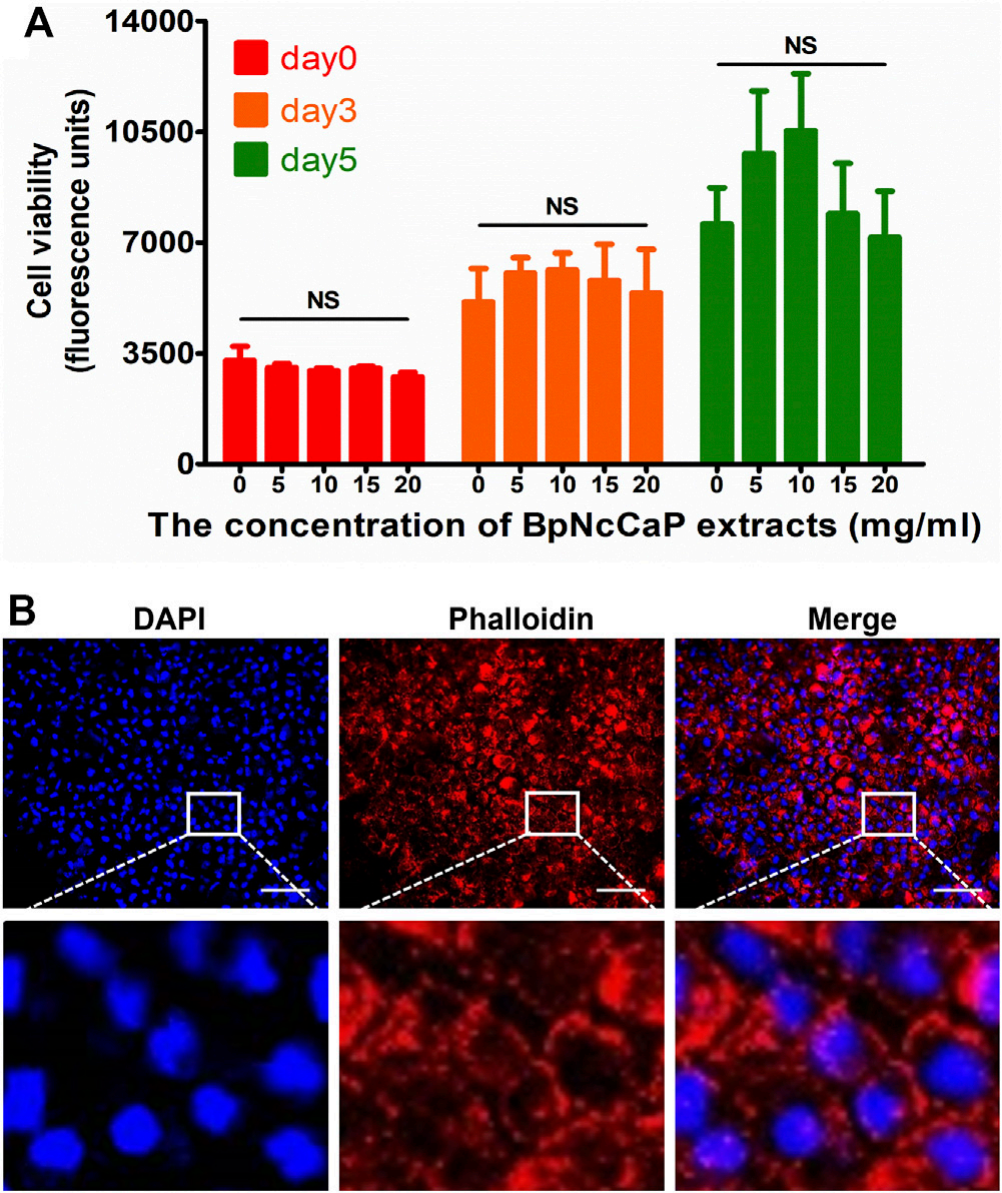
Graph depicting the metabolic activity of MC3T3-E1 exposed to the different concentrations of BpNcCaP extracts using a PrestoBlue assay **(A)**. Fluorescent micrographs showing the nuclei (blue) and actin filaments (red) of MC3T3-E1 pre-osteoblasts cultured on BpNcCaP discs **(B)**. The cells were cultured for 3 days and then fixed and stained with rhodamine-phalloidin and DAPI. Bar = 100 μm.

PrestoBlue assay was adopted to assess the cytotoxicity of BpNcCaP. As shown in Figure 6A, the metabolic viability (fluorescence units) of the MC3T3-E1 cells of three groups (5, 10, 15 mg/ml of BpNcCaP extracts) increased gradually with the culture time. The cell viability was also the highest in the group of 10 mg/ml of BpNcCaP extracts, while although no significant difference was found among all the groups at each culture time point. This result indicated that the BpNcCaP has no obvious cytotoxicity. We further adopted Rhodamine-Phalloidin and DAPI to stain the actin and nuclei of attached cells as an indicator for the cytocompatibility of BpNcCaP. As shown in Figure 6B, after 3 days post-seeding, MC3T3-E1 pre-osteoblasts spread on the BpNcCaP discs with a high density. This result indicated that the BpNcCaP was favorable for the adhesion, spreading and proliferation of osteogenic cells.

### 3.3 BMP-2 Dosage in BpNcCaP

Using the ELISA kit, the internally-incorporated BMP-2 dosage in BpNcCaP was determined and shown in Table 1. The amounts of BMP-2 in the groups of BpNcCaP+5 μg BMP-2, BpNcCaP+10 μg BMP-2, BpNcCaP+25 μg BMP-2 and BpNcCaP+50 μg BMP-2 were 4.31 ± 0.17 μg, 9.39 ± 0.14 μg, 23.06 ± 2.19 μg and 52.03 ± 4.81 μg, respectively. The corresponding loading efficiencies were 43.08 ± 1.67%, 46.97 ± 0.68%, 57.65 ± 5.47% and 65.04 ± 6.01%, respectively.

### 3.4 Micro-CT Analysis

**FIGURE 7:**
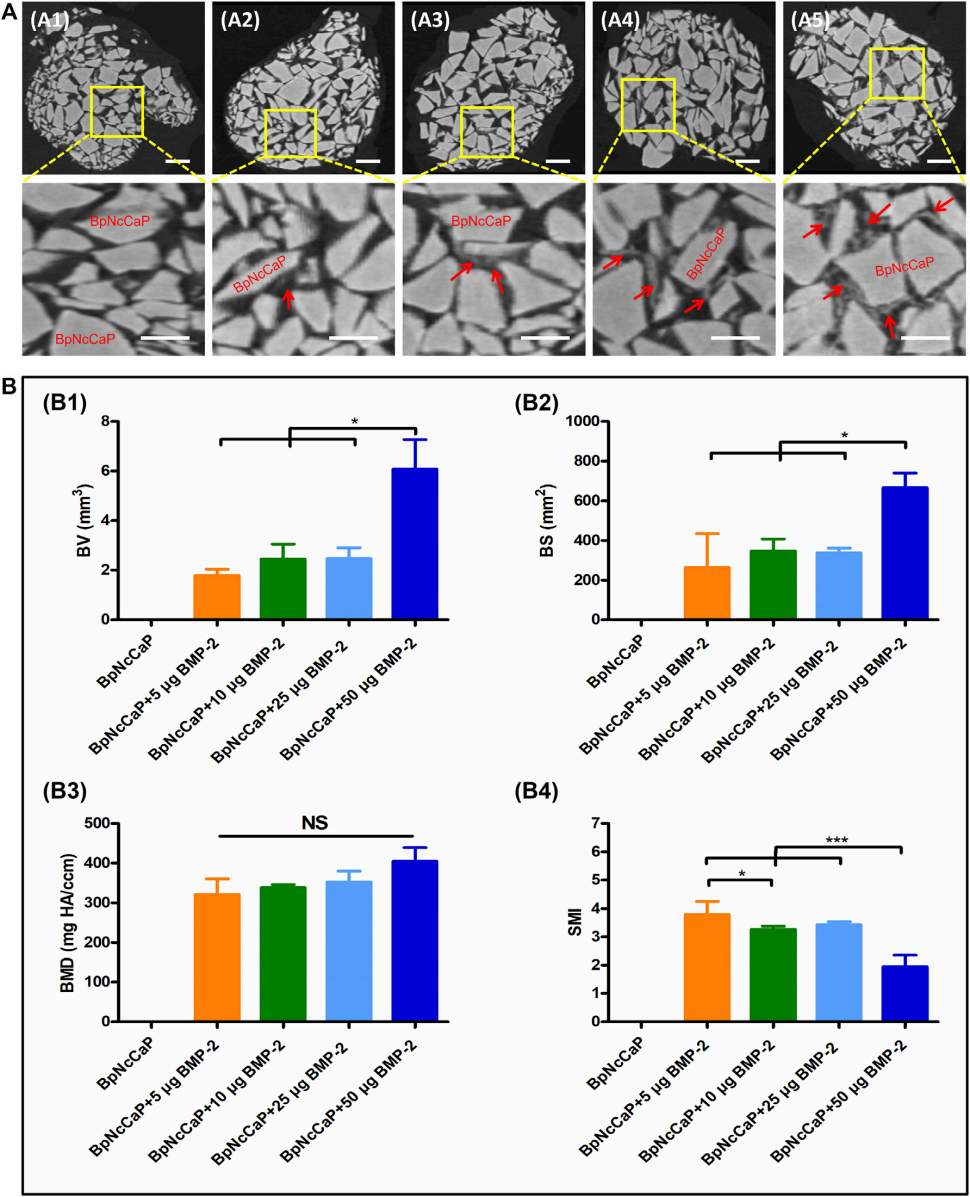
Micro-CT images **(A)** showed newly formed bone and grafted biomaterials of BpNcCaP without **(A1)** or with different amount (5/10/25/50 μg) of BMP-2 **(A2–A5)** 5 weeks post implantation. White granules: BpNcCaP; Red arrow: new bone tissue. Bar = 1 mm. Micro-CT analysis **(B)** of the new bone volume **(B1)**, bone surface area **(B2)**, bone mineral density **(B3)**, structure mode index **(B4)** for BpNcCaPs without or with different amounts (5/10/25/50 μg) of BMP-2. Data were presented as mean ± SD. Significant effect of the treatment, *p < 0.05; **p < 0.01; ***p < 0.001.

We adopted micro-CT analysis to qualitatively and quantitively evaluate the new bone formation surrounding BpNcCaP without and with BMP-2 incorporation (Figure 7). Micro-CT images (Figure 7A) showed that newly formed bone (red arrows) occurred on BpNcCaP granules or in the intra-granular space. More newly formed bone was detected with the increase of BMP-2 incorporation amount. No new bone was found in the group of BpNcCaP without BMP-2. The total bone volume (BV) (Figure 7B1) in the group of BpNcCaP+50 μg BMP-2 was significantly higher than those of the other BMP-2 containing groups. The same pattern was also found for BS (Figure 7B2). The SMI (Figure 7B4) in the group of BpNcCaP+50 μg BMP-2 was significantly lower than those of the other BMP-2-containing groups. No significant difference was found in BMD (Figure 7B3) among the BMP-2-containing groups.

### 3.5 Histological and Histomorphometric Analysis

**FIGURE 8:**
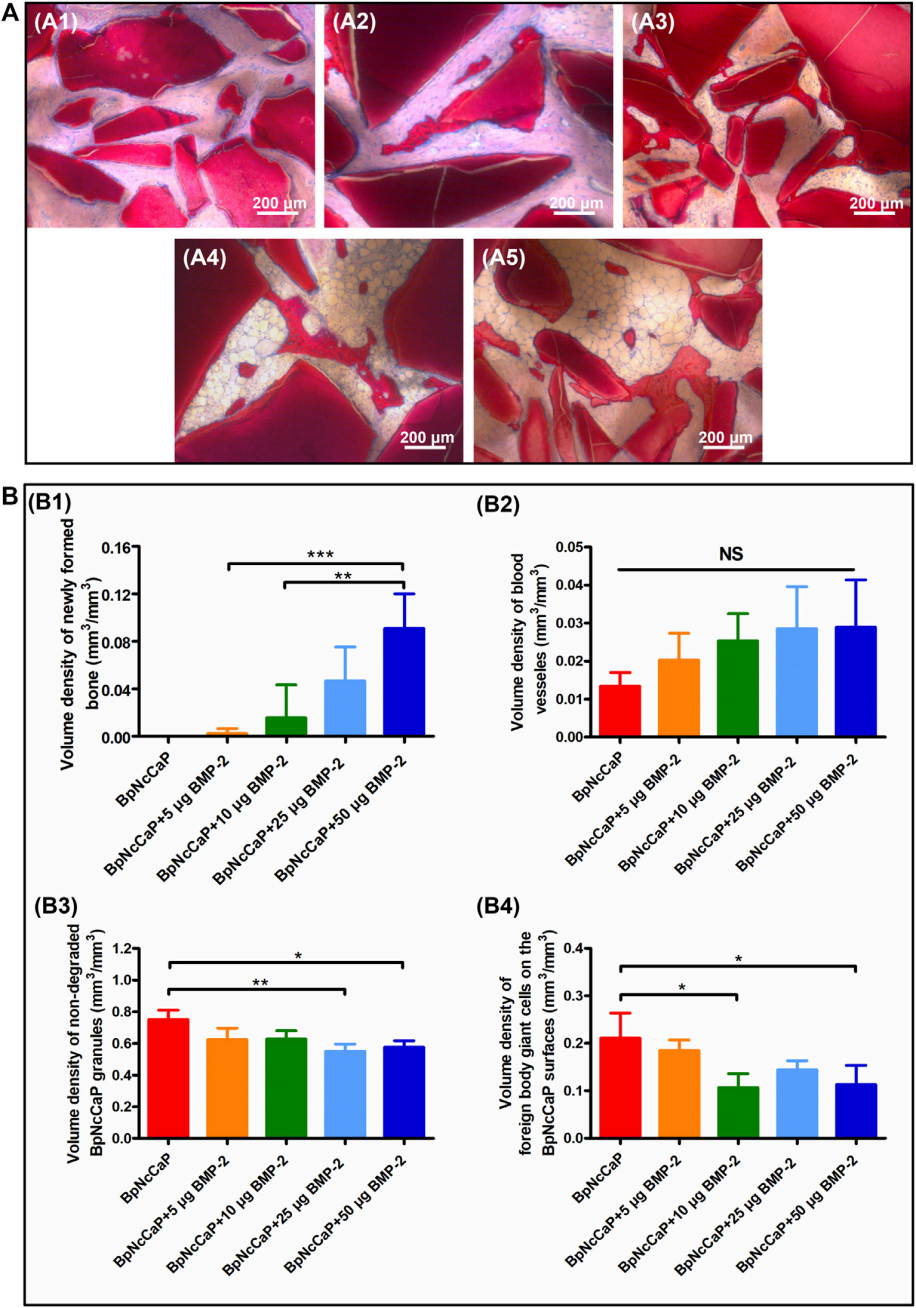
Light micrographs **(A)** of newly formed bone and remaining BpNcCaP without **(A1)** or with different amount (5/10/25/50 μg) of BMP-2 **(A2-A5)** 5 weeks after implantation. Graphs **(B)** depicting the volume densities of newly formed bone **(B1)**, blood vessels **(B2)**, undegraded particles **(B3)** and foreign body giant cells **(B4)** on the surfaces of BpNcCaP and 5/10/25/50 μg BMP-2 incorporated BpNcCaP granules. The sections were surface-stained with McNeal’s Tetrachrome, basic Fuchsine and Toluidine Blue O. Mean values (*n* = 6) were represented together with the standard deviation, significant effect of the treatment: *p < 0.05; **p < 0.01; ***p < 0.001.

**FIGURE 9:**
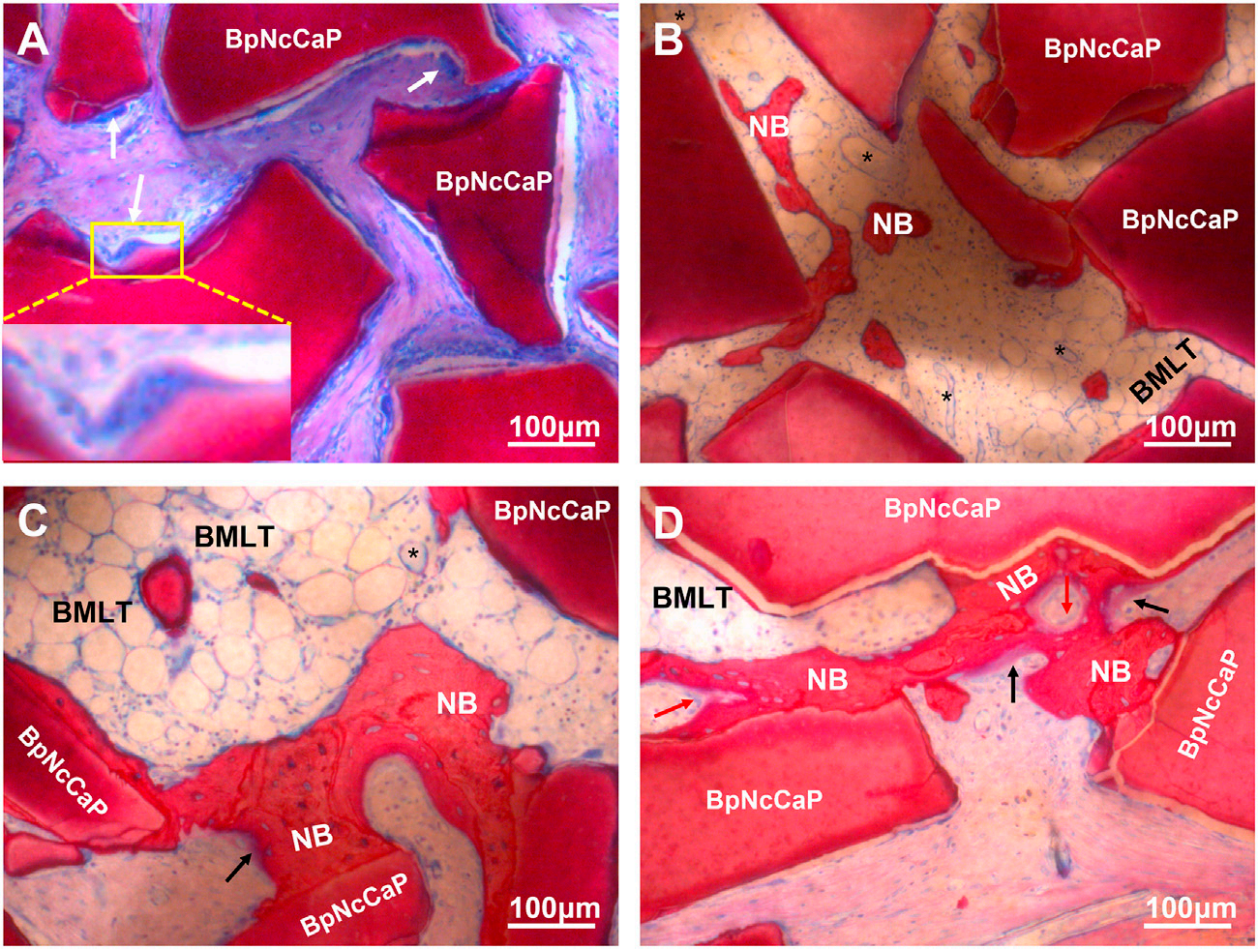
Light micrographs in high magnification of BpNcCaP **(A)** and BpNcCaP+50 μg BMP-2 **(B–D)** 5 weeks after subcutaneous implantation in rats. NB: New bone; Black asterisk: Blood vessels; Black arrow: Unmineralized bone areas; White arrow: Foreign body giant cells (FBGCs); Red arrow: Osteoblasts; BMLT: Bone marrow-like tissues.

Five weeks after the subcutaneous implantation, no new bone formation was detected in the group of BpNcCaP without BMP-2 incorporation (Figures 8A1, 9A). Instead, a large amount of foreign body giant cells (FBGCs) (Figure 9A) were found along the surfaces of BpNcCaP. In the group of BpNcCaP+5 μg BMP-2 (Figure 8A2), only very little bone occurred and distributed sporadically in the intra-granular space. In the group of BpNcCaP+10 μg BMP-2 (Figure 8A3), many bone tissues aligned along the surfaces of BpNcCaP granules and extended to the adjacent BpNcCaP granules, which formed an integrated network. In the groups of BpNcCaP+25 μg BMP-2 (Figures 8A4, 50 μg BMP-2 (Figures 8, 9B–D), many bone marrow-like tissues (BMLT) occurred in the intra-granular space with newly formed bone tissue as borders. Newly formed osteoid tissues with purple color were more frequently found aligned on mineralized bone tissue. On the osteoid tissue, there was a layer of uniformly aligned osteoblasts with cuboid or cube-like morphology. Less FBGCs were detected with the increase of BMP-2 incorporation amount. Quantitative analysis (Figure 8B) showed an increasing trend of volume density of newly formed bone (Figure 8B1) with the increase of incorporated BMP-2 amount. The volume density of newly formed bone in the group of BpNcCaP+50 μg BMP-2 was significantly higher than those in the groups of BpNcCaP+5 μg BMP-2 and BpNcCaP+10 μg BMP-2. The volume density of blood vessels (Figure 8B2) was also the highest in the group of BpNcCaP+50 μg BMP-2 although no significant difference was found among all the groups. In comparison with BpNcCaP without BMP-2, the volume densities of BpNcCaP material (Figure 8B3) in the groups of BpNcCaP+25 μg BMP-2 and BpNcCaP+50 μg BMP-2 were significantly decreased. The volume density of FGBCs (Figure 8B4) showed a decreasing trend with the increase of BMP-2 incorporation amount with the lowest value detected in the group of BpNcCaP+50 μg BMP-2.

## 4 Discussion

Most of the clinically available bone-defect-filling materials lack proper degradability and efficient osteoinductivity (Bhat et al., 2021). In this study, through greatly improving our previous biomimetic precipitation protocol, we synthesized novel BpNcCaP granules with internally incorporated BMP-2. *In-vitro* characterization data showed that the BpNcCaP + BMP-2 granules were comprised of hexagonal HA with an average crystallite size ranging from 19.7 to 25.1 nm, a crystallinity ranging from 91.12 to 94.07% and a grain size at 84.13 ± 28.46 nm. The vickers hardness of BpNcCaP was 32.50 ± 3.58 HV 0.025. BMP-2 incorporation rate could even reach 65.04 ± 6.01%. BpNcCaP showed no obvious cytotoxicity and was favorable for the adhesion of pre-osteoblasts. *In-vivo* histomorphometric analysis showed that the total volume of new bone induced by BpNcCaP exhibited a BMP-2 amount-dependent increasing manner. The BpNcCaP+50 μg BMP-2 exhibited significant degradation and less FBGCs reaction in comparison with BpNcCaP. These data suggested a promising application potential of BpNcCaP + BMP-2 in repairing LVBDs.

In the family of CaP, one of the most relevant materials for bone regeneration is HA due to its bone-like composition and thus excellent biocompatibility and osteoconductivity (Bhat et al., 2021). One method to produce HA-based bone-defect-filling materials for clinical application is to harvest natural HA from allogenic bone tissues (Ramesh et al., 2018), which involves various decellularization processes, such as washing using enzyme, acetone or supercritical CO_2_ (Crapo et al., 2011; Ha et al., 2013; Chalard, 2021; Jiang et al., 2021). Since the organic compositions are not completely removed, their usage is associated with a potential risk of severe immune reaction and rejection (Li et al., 2014). Xenogeneic grafts, such as Bio-Oss^®^ or Cerabone^®^ are conventionally further processed by sintering at 300 or even 1,250°C for at least several hours to remove all organic compositions, which confers them excellent biocompatibility (Barbeck et al., 2017). However, such a sintering causes a significant increase in crystallinity of HA and thus a dramatic decrease of degradation, hindering natural bone replacement. Many *in-vivo* studies have proved that even after 9 months of implantation sintered HA remained at the implantation sites with hardly any sign of resorption (Klein et al., 1983; Okuda et al., 2008; Diez-Escudero et al., 2017). Synthetic HA for fabricating bone-defect-filling materials is usually achieved via wet chemical precipitation, where calcium and phosphate precursors are mixed with ammonium hydroxide (NH_4_OH) as a precipitation agent (Massit et al., 2018; López-Ortiz et al., 2020; Nimishakavi et al., 2021). The thereby-achieved HA is in fine powder and still needs to be sintered at 600–1,300°C for 1–48 h, where the grain size is significantly increased to facilitate the formation of granules with sufficient stiffness (Champion, 2013; Rodríguez-Lugo et al., 2018). Therefore, the disadvantages of sintered HA, such as low degradability and high hardness pursues (Zhou et al., 2018; Sikder et al., 2020). Furthermore, bioactive agents, such as proteinous growth factors (e.g., BMP-2) can only be adsorbed on to these materials, which exhibits too burst a release to continuously sustain bone regeneration (Agrawal and Sinha, 2017). Such a problem is hardly solved by simply adding polymeric materials since regulatory difficulties will be greatly increased. Consequently, it is in a great need to develop a novel fabrication method to produce HA-based bone-defect-filling materials with slow delivery, proper degradability, and stiffness. To provide a viable option, following a biomimetic principle, we have already developed a new type of CaP granules with a slow delivery property and cancellous bone-like stiffness (Zheng et al., 2014; Liu et al., 2017; Wang et al., 2017). However, the thereby-produced CaP granules are still suboptimal due to the simultaneous incorporation of chemicals, such as Tris base and sodium chloride, which may compromise the biocompatibility of the granules and the osteoinductive efficacy of BMP-2 (Konar and Sahoo, 2019). Furthermore, the incorporation rate of BMP-2 is also low (about 20–30%) (Wu et al., 2011; Wang et al., 2017), which greatly increases the cost of the products. In this study, we improved the fabrication method to synthesize novel biomimetic CaP granules with minimized presence of the unintended chemicals and maximal incorporation rate of BMP-2.

One critical step in this novel fabrication method was to substitute BMP-2 co-precipitation with washing the precipitates using PBS before the addition of BMP-2, which was highly important to remove the unintended chemicals. We adopted EDS to evaluate the efficiency of washing with PBS in eliminating residual chemicals using Na element as an indicator. We found that wt% of Na element was as high as 33.86%, which indicated the abundant presence of NaCl in the granules before washing (Supplementary Figure S2). After washing for 5 times, wt% of Na element dramatically decreased to 1.34%, which suggested that most of the unintended chemicals were removed. In SEM, we found that BpNcCaP granules bore a rough and slightly uneven morphology. High-magnification SEM images showed that BpNcCaP was mainly comprised of needle/rod-like crystals with the grain length at 85.14 ± 24.48 nm. FTIR analyses showed that BpNcCaP contained the chemical groups of PO_4_^3−^, CO_3_^2-^, OH^−^. The XPS survey spectra showed that BpNcCaP granules that contained the photoelectron peaks belonging to calcium (Ca), phosphorus (P) and oxygen (O) were consistent with XPS results reported for Ca 2p, P 2p, and O 1s in HA (Rojas-Mayorga et al., 2016; Gomes et al., 2017; Rodríguez-Lugo et al., 2018; Senthilkumar et al., 2021). In addition, the presence of CO_3_^2-^ suggested that our BpNcCaP was at least partially carbonated, which may increase its bioactivity (Zapanta-Legeros, 1965; Barralet et al., 2002; Diez-Escudero et al., 2017). XRD analysis showed that the main chemical composition of BpNcCaP was hexagonal HA according to the ICSD-PDF card 86-0740. The unit cell parameters (a = b = 0.946 nm and c = 0.687 nm) of BpNcCaP resembled the characteristic parameters of HA (Vargas-Becerril et al., 2020). In conventional methods to fabricate granular bone-defect-filling materials, sintering is always adopted to increase the grain size, during which the crystallite size of HA also dramatically increases to 45–55 nm (Rodríguez-Lugo et al., 2018). A study by Liu et al. found high degrees crystallinity of sintered HA was 95–99% at heating temperatures between 600 and 1,000°C (Liu et al., 2015). Furthermore, the crystallinity of sintered HA was increased by high temperature, which resulted in significantly decreased degradability (Diez-Escudero et al., 2017; Safarzadeh et al., 2020). In our granules, the average crystallite size of BpNcCaP was kept as small as 24.6 nm since no sintering was adopted in our biomimetic production process. Albeit so, the grain size of the BpNcCaP was also increased to 85.14 ± 24.48 nm, which ensured the formation of granular form. Furthermore, the crystallinity was also kept as low as 92%, which contributed to a proper degradation. To our best knowledge, this was the first report to reveal the physicochemical properties of biomimetically produced CaP granules. Furthermore, these physicochemical properties were not significantly changed by the presence of BMP-2 irrespective of its amount, which was also reasonable since BMP-2 was not co-precipitated but added after precipitation, washing and condensation.

Another advantage of this novel method was the significantly enhanced incorporation rate of BMP-2. BMP-2 is an expensive bioactive agent with about several thousand euros per 12 mg. Therefore, enhancing the incorporation rate is highly important to reduce the cost of BpNcCaP + BMP-2, which is beneficial for its wide-spreading in clinic. Our previous study shows that, using our biomimetic coating procedure to functionalize a xenogenic deproteinized bovine bone, the incorporation rate of BMP-2 is about 20.0 ± 0.7% (Wu et al., 2011). When using our previous biomimetic precipitate with the co-precipitation of BMP-2, the incorporation rate of BMP-2 is about 30.1 ± 5.7% (Liu et al., 2017; Wang et al., 2017). For both methods, BMP-2 is added into a large volume of the biomimetic solution to facilitate the co-precipitation with CaP, during which a large portion of BMP-2 maintains in the solutions, thereby resulting in a low incorporation rate. In comparison, in this novel method, we added BMP-2 into a much smaller volume of the collected, washed and centrifuged precipitates, which significantly reduced the waste of BMP-2 during removing the much smaller volume of solution. We showed that the incorporation rate of BMP-2 of BpNcCaP was 43.08 ± 1.67 when 4.31 ± 0.17 μg/sample BMP-2 was incorporated and could be enhanced to as high as 65.04 ± 6.01% when 52.03 ± 4.81 μg BMP-2 per gram BpNcCaP was incorporated. These incorporation rates were already 1.4 to 2.2 times of that for the granules produced using our previous methods, which was highly meaningful to enhance its clinical applicability.

Thereafter, we adopted the subcutaneous bone induction model (Liu et al., 2005; Wu et al., 2011) to assess the osteoinductive efficacy of the BpNcCaP granules with different amounts of incorporated BMP-2. We first adopted micro-CT to quantitatively and qualitatively evaluate the new bone formation 5 weeks post-implantation, the BpNcCaP without BMP-2 did not induce any new bone formation. New bone formation occurred in the group of BpNcCaP+5 μg BMP-2 and the BVs were similar in the groups of BpNcCaP+10 μg BMP-2 and BpNcCaP+25 μg BMP-2. In comparison, BV in the group of BpNcCaP+50 μg BMP-2 was significantly higher than those in the other three BMP-2-containing groups. The same pattern was also found in BS. These data indicated the bone-inducing capacity of BpNcCaP+50 μg BMP-2 was much stronger than the other granules. SMI is the quantification of the balance between rod-like and plate-like trabecular structures. The SMI value is 0 for an ideal plate structure, and 3 for a perfect rod structure or an infinite circular cylinder; round spheres have an SMI of 4 (Jiang et al., 2005). The SMI in the group of BpNcCaP+50 μg BMP-2 was significantly lower than that in other groups, which suggested that the trabecular structure of BpNcCaP+50 μg BMP-2 became thicker and denser after bone remodeling. While no significant difference was found in BMD, which suggested that the quality of new bone induced by BpNcCaPs with different amounts of BMP-2 was similar to each other. These data indicated that BpNcCaP+50 μg BMP-2 bore a significantly higher osteoinductive capacity, resulting in not only significantly enhanced bone quantity but also optimized bone microstructures.

We adopted the non-demineralized (hard) tissue sectioning technique to analyze the tissue reactions surrounding BpNcCaP without or with different mounts (5, 10, 25, 50 μg) of internally incorporated BMP-2 as we previously reported (Hägi et al., 2010; Wu et al., 2011). The advantages of this technique over a demineralized tissue sectioning are the maximal preservation of mineralized phases, including bone tissues and CaP-based bone grafts, thus their interfaces (Erben, 1997). With the aid of this staining, we can histomorphometrically analyze and stereologically quantify volume densities of bone, blood vessels and FBGCs (Wu et al., 2010b; Wu et al., 2011; Liu et al., 2017). Histological staining showed that the volume density of newly formed bone in the group of BpNcCaP+50 μg BMP-2 was significantly higher than those in the groups of BpNcCaP+5 μg BMP-2 and BpNcCaP+10 μg BMP-2. This phenomenon indicated that BpNcCaP+50 μg BMP-2 bore the most osteogenic capacity. Another important parameter for bone regeneration is the formation of blood vessels. Blood vessel is important for providing nutrients to osseous tissue. Moreover, blood vessels also release paracrine signals to modulate the growth, differentiation and regeneration of bone cells (Diomede et al., 2020). Our data showed that the volume density of blood vessels exhibited an increasing trend with the increasing loading of BMP-2. The average volume density of blood vessels was about 2.2 times that in BpNcCaP, suggesting that BMP-2 also promoted the formation of blood vessels accompanying its-induced osteogenesis although no significant difference was found among all the groups. In previous studies, co-administration of angiogenic bioactive agents, such as hyaluronic acid and human salivary histatin-1 with BMP-2 on absorbable collagen sponge lead to significantly enhanced angiogenesis and osteogenesis (Huang et al., 2017; Sun et al., 2020). Consequently, we may further improve the angiogenic and osteogenic efficacy of BpNcCaP + BMP-2 by simultaneously internally incorporating these materials. The volume density of FBGCs showed a decreasing trend with the increase of BMP-2 incorporation amount with the lowest value detected in the group of BpNcCaP+50 μg BMP-2. The foreign body reaction is mediated by monocytes, macrophages and FBGCs (Sheikh et al., 2015; Martin and García, 2021). When monocytes/macrophages can hardly resorb large foreign body objectives, they can fuse into multinucleated FBGCs to enhance their resorbing efficacy. Therefore, FBGCs are regarded as a marker of chronic inflammation and foreign-body reaction (Wu et al., 2011; Liu et al., 2013; Liu et al., 2017). Therefore, the lowest volume density of FBGCs in the group of BpNcCaP+50 μg BMP-2 suggested a higher biocompatibility and less risk of immune rejection than BpNcCaP.

In our previous study, we find that biomimetically precipitated CaP coating is resorbed much faster when extensive bone formation was induced by its incorporated BMP-2 than that when no new bone formation occurs due to the absence of BMP-2 (Wu et al., 2010b). Thereafter, we further show a similar phenomenon for our biomimetically layer-by-layer (alternate amorphous CaP and crystalline CaP) assembled CaP granules (Zheng et al., 2014). It seems that the degradation of biomimetic CaP coating-based material shows a bone formation-responsive property, e.g., the more new bone formation and the more degradation of biomimetic CaP coating-based materials. This property may be largely attributed to the absence of sintering during its production process. In this study, our BpNcCaP also inherited such a bone formation-responsive property, which was evidenced by the decreasing trend of remaining BpNcCaP with the increase of new bone at 5 weeks post-implantation. Such a property of BpNcCaP granules holds a dramatic contrast to sintered HA that barely degrades even after an implantation for several months (Okuda et al., 2008; Diez-Escudero et al., 2017). This property of BpNcCaP is highly beneficial for both space preservation before bone ingrowth and the subsequent substitution of these bone-defect-filling materials with host bone tissue. In addition, the more bone formation was also associated with the less volume density of FBGCs. This was consistent with our previous finding that volume density of new bone is negatively correlated with the volume density of FBGCs. This might be largely due to the reduced direct exposure of BpNcCaP to connective tissues and thus immune system when more new bone formed on the surface of BpNcCaP, thereby causing less foreign-body reaction.

One limitation in this study was that we adopted an ectopic bone induction model to evaluate the osteogenic efficacy of BpNcCaP + BMP-2. Further studies can be performed to evaluate its performance in orthopedic bone defects. Furthermore, large animal studies should also be performed before extrapolating the present data to clinical application.

## 5 Conclusion

In this study, we synthesized novel BpNcCaP granules with internally incorporated BMP-2 with an aim to develop properly degradable and highly osteoinductive granules to repair LVBDs. *In-vitro* characterization data showed that the BpNcCaP + BMP-2 granules were comprised of hexagonal HA with an average crystallite size ranging from 19.7 to 25.1 nm and a grain size at 84.13 ± 28.46 nm. The vickers hardness of BpNcCaP was 32.50 ± 3.58 HV 0.025. BpNcCaP showed no obvious cytotoxicity and was favorable for the adhesion of pre-osteoblasts. BMP-2 incorporation rate could even reach 65.04 ± 6.01%. *In-vivo* histomorphometric analysis showed that total volume of new bone induced by BpNcCaP exhibited a BMP-2 amount-dependent increasing manner. The BpNcCaP+50 μg BMP-2 exhibited significant degradation and less FBGCs reaction in comparison with BpNcCaP. These data suggested a promising application potential of BpNcCaP + BMP-2 in repairing LVBDs.

## Data Availability

The original contributions presented in the study are included in the article/Supplementary Material, further inquiries can be directed to the corresponding authors.
