# Supplementary material for: Development, In-Vitro Characterization and In-Vivo Osteoinductive Efficacy of a Novel Biomimetically-Precipitated Nanocrystalline Calcium Phosphate With Internally-Incorporated Bone Morphogenetic Protein-2
Source: Front Bioeng Biotechnol. 2022 Jul 22;10:920696. doi: 10.3389/fbioe.2022.920696 (PMC9354744; doi:10.3389/fbioe.2022.920696)
Supplement: Supplementary file 1 [file DataSheet1.pdf]

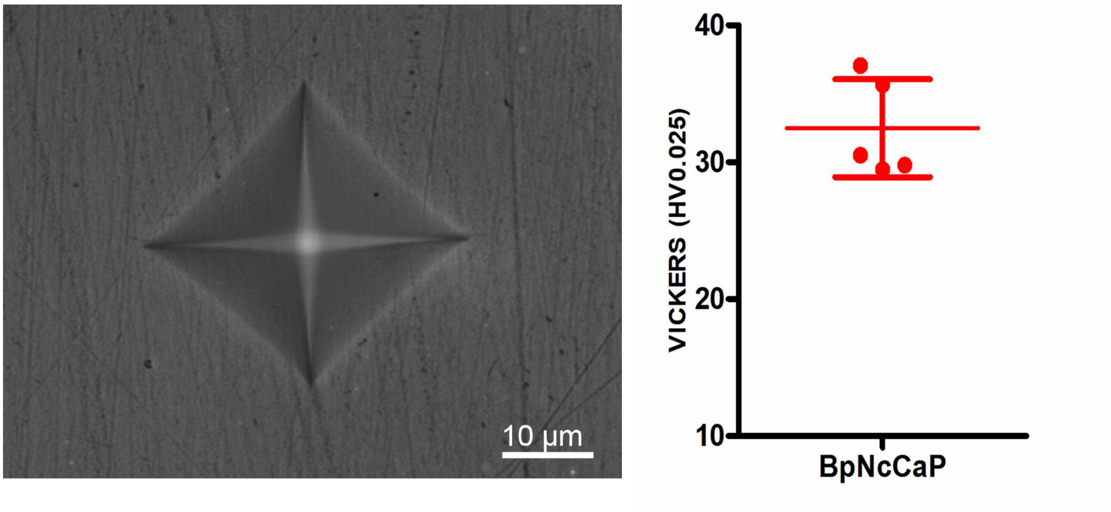

Supplementary figure 1. The vickers hardness of BpNcCaP.

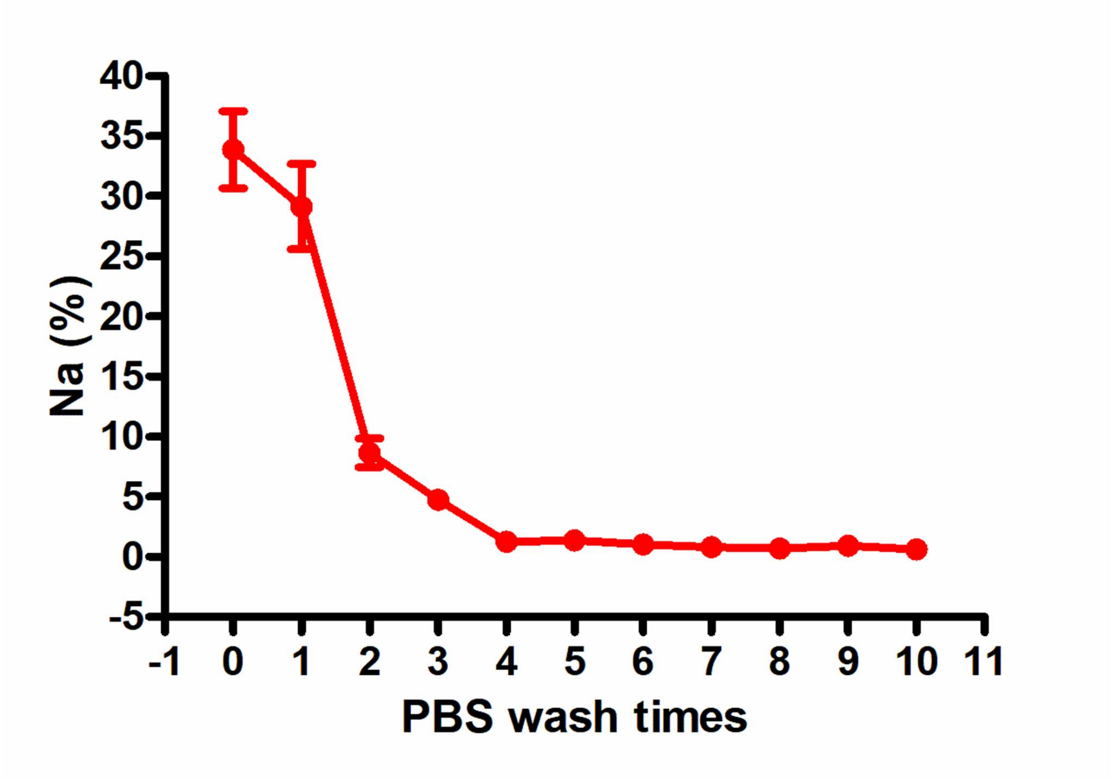

Supplementary figure 2. Residual Wt% of Na element of BpNcCaP precipitation after PBS wash times.
